# Supplementary material for: Leishmania tarentolae and Leishmania infantum in humans, dogs and cats in the Pelagie archipelago, southern Italy
Source: PLoS Negl Trop Dis. 2021 Sep 23;15(9):e0009817. doi: 10.1371/journal.pntd.0009817 (PMC8491888; doi:10.1371/journal.pntd.0009817)
Supplement: Attachment — Submitted filename: Authors response.docx [file pntd.0009817.s001.docx]

## Dear Dr. Otranto,

## Thank you very much for submitting your manuscript "*Leishmania tarentolae* and *Leishmania infantum* in humans, dogs and cats in the Pelagie archipelago, southern Italy" for consideration at PLOS Neglected Tropical Diseases. Apologies for the delay in sending a decision, but we had a delay finding suitable reviewers for your work. As with all papers reviewed by the journal, your manuscript was reviewed by members of the editorial board and by several independent reviewers. In light of the reviews (below this email), we would like to invite the resubmission of a significantly-revised version that takes into account the reviewers' comments.

## We cannot make any decision about publication until we have seen the revised manuscript and your response to the reviewers' comments. Your revised manuscript is also likely to be sent to reviewers for further evaluation.

**Authors’ response:**

## First of all, I would like to thank you for your editorial work. I fully understand the difficulties you faced in identifying the reviewers. Considering the unexpected long time since the first submission of the manuscript, I would be grateful you could guarantee a fast second round of revision for our submission.

## We considered all comments raised and carefully reviewed the entire manuscript accordingly as reported in our point-to-point reply.

Reviewer's Responses to Questions
**Methods**
-Are the objectives of the study clearly articulated with a clear testable hypothesis stated?
-Is the study design appropriate to address the stated objectives?
-Is the population clearly described and appropriate for the hypothesis being tested?
-Is the sample size sufficient to ensure adequate power to address the hypothesis being tested?
-Were correct statistical analysis used to support conclusions?
-Are there concerns about ethical or regulatory requirements being met?

**Reviewer #1**: All clear.
**Reviewer #2**: It is not clear how the sizes of human, canine and feline populations were determined.
**Authors’ response:**

The number of human sample size was based on the eagerness of individuals to participate in the study upon signing a consent form. The same for pet owners enrolling their animals.

**Reviewer #3**: In this manuscript authors show interesting results of a potentially new *Leishmania* transmission scenario, involving a parasite from the subgenus Sauroleishmania. However, in order to strengthen the findings, more robust methods should be used. 
Some relevant questions:
Can you perform an identification of the L. tarentolae strain (TAR and LEM) to provide further evidence of the infection potential to human hosts?

**Authors’ response:**

Thank you for this valuable comment. The *L. tarentolae* strain used in this study has been identified by Istituto Superiore di Sanità, that is the reference laboratory for *Leishmania* in Italy. As requested we added the strain number (LEM124).

How specific are the serological tests used for *L. tarentolae* screening? Can you include other *Leishmania* species in your IFATS to account for inespecificities?

**Authors’ response:**

The IFAT technique is based on the whole promastigotes of both *L. infantum* and *L. tarentolae*, two species genetically very similar. The occurrence of a cross-reactivity may be possible, which represents a limitation of the test (see discussions). As mentioned in the text, the sensitivity and specificity of this serological method should be evaluated by comparing it with a more accurate test such as Western blot. In addition, although cross-reactions may occur with highly similar species of *Leishmania*, the patients and animals enrolled in this study were autochthonous without a travel history abroad. Therefore, considering the epidemiology of leishmaniasis in the Mediterranean basin the risk of infection by other species is unlikely. The text has been amended accordingly.

--------------------

**Results**
-Does the analysis presented match the analysis plan?
-Are the results clearly and completely presented?
-Are the figures (Tables, Images) of sufficient quality for clarity?

**Reviewer #1**: Lines 208-210: were these the same samples? meaning, were the serologically positive individuals also PCR-positive?

**Authors’ response:**

Out of 23 human samples positive to *L. tarentolae*, 12 were only seropositive and 11 only molecularly. This has been clarified in the text.

lines 217-219: please, also give the number of base pairs (lengths of the respective fragments)

**Authors’ response:**

Done.

line 240: why 53.5%? how does this correlate to data given in table 2?
**Authors’ response:**

53.5% is the cumulative prevalence of dogs exposed to *L. tarentolae* or *L. infantum* only, and to both species.

Reviewer #2: Comparison between serological and molecular results could have been made.
**Authors’ response:**

The Authors calculated the agreement between serological and molecular tests for the detection of antibodies or DNA of both *Leishmania* spp. and added it in the text.

Reviewer #3: ITS based analyses are characterized by a short fragment, which can lead to inespecificities. To strengthen the results, authors could include a marker with a longer fragment for the detection of *L. tarentolae*. In this way, they could perform comparative analysis, even including other Leishmania species. HSP70 has already been used in *L. tarentolae* and it generates larger fragments, so there are good reference bases. Also including a tree would be very helpful. 
Regarding serological tests as the basis for the diagnosis of these two Leishmania species, the specificity of the test against *L. tarentolae* is not clear, again, the use of other Leishmania species as reference would be desirable.

**Authors’ response:**

The authors agree with the reviewer’s comment on the short fragment of ITS1 analyzed. However, the specificity of the *Leishmania* spp. detected is given by the use of the dqPCR based on species-specific probes for *L. infantum* and *L. tarentolae*. The sequence analysis, of random samples, has been considered as supportive of species identification. Furthermore, considering the aim of the study we consider that the HSP70 gene and/or a phylogenetic analysis do not add useful information.

Regarding serological tests, please see comment above.
--------------------

**Conclusions**
-Are the conclusions supported by the data presented?
-Are the limitations of analysis clearly described?
-Do the authors discuss how these data can be helpful to advance our understanding of the topic under study?
-Is public health relevance addressed?

Reviewer #1: line 259: except those with detectable DNA (how does this correlate to antibody titers and any known immunodeficiency?).

**Authors’ response:**

For the seek of clarity, the text has been reworded accordingly.

line 268: how does your study prove long-lasting contact with reservoirs and why is this important?

**Authors’ response:**

We probably over discussed the results in the attempt to explain them. The sentence has been deleted.

line 283: rephrase, as this would imply that DNA detection was possible already in the 18th century.

**Authors’ response:**

Done.

line 326: this "potential beneficial role" should be explained and discussed in more detail in the Discussion.

**Authors’ response:**

Done.

Reviewer #2: Conclusions are those of a preliminary study. Cross-reactivity of the serological tests could have been addressed.

**Authors’ response:**

The authors modified the conclusions highlighting the limitations of the IFAT.

Reviewer #3: Although authors are conscious of the limitations of their study, evidence for public health relevance is missing and could benefit from additional experiments and analysis.
**Authors’ response:**

Thank you for your comment. This concept has been better addressed and further discussed.

--------------------

**Editorial and Data Presentation Modifications?**
Use this section for editorial suggestions as well as relatively minor modifications of existing data that would enhance clarity. If the only modifications needed are minor and/or editorial, you may wish to recommend “Minor Revision” or “Accept”. 

Reviewer #1: Numerous minor grammatical errors and typos, some examples are given below.
lines 31-32: "by multiple serological and molecular testing" should go behind the bracket in line 32.
line 35: should be: sera, and: tested for
line 75: this should be "immunocompromised", I assume?
line 82: should be: *Sergentomyia* and *perniciosus*

**Authors’ response:**

The grammatical errors and typos have been corrected throughout the manuscript.

Reviewer #2: (No Response)

Reviewer #3: (No Response)

--------------------

**Summary and General Comments**

Use this section to provide overall comments, discuss strengths/weaknesses of the study, novelty, significance, general execution and scholarship. You may also include additional comments for the author, including concerns about dual publication, research ethics, or publication ethics. If requesting major revision, please articulate the new experiments that are needed.

Reviewer #1: This is an interesting study with several novel aspects (detection of *L. tarentolae* and corresponding antibody response in humans and pets), but it could easily be condensed to a Short Report.
**Authors’ response:**

Given the relevance of the results in the field and for avoiding to delete important data and comments, the authors would prefer to present the study as a research article.

Reviewer #2: Line 30 – change to: *Leishmania tarentolae* WAS detected
Line 42 – *Leishmania* spp. – change accordingly throughout the manuscript

Line 52 – change to: This species, non-pathogenic to mammals, has recently been…
Line 62 – change to: With over 20 Leishmania spp. as causative agents, THE leishmaniases are neglected … tropical diseases
Line 67 – add comma: most important species of zoonotic concern, with domestic dogs
Line 68 – change to: The Mediterranean basin is A hyperendemic area
Line 70 – change to: immunocompetent hosts, ARE 5–10 times greater 
Line 77 – change to: Tyrrhenian and the low Adriatic Coasts and ON the islands, according to the distribution of the sand
Line 79 – centre
Line 81 – parasites (instead of parasite)
Line 90 – change to: In addition, this species, non-pathogenic to humans, has…
Lines 93-96 – sampling of dogs and cats should also be mentioned

**Authors’ response:**

Done.

Line 122 – which criteria (statistical, etc.) have determined these samples sizes?
**Authors’ response:**

The number of human sample size was based on the eagerness of individuals to participate in the study upon signing a consent form. The same for pet owners enrolling their animals.

Line 123 – change to: and n=101 from Linosa) without any specific sign of leishmaniasis, were COLLECTED and sent to the
Line 126 – were purposely collected samples (under the frame of this study, for health check analyses) all from Linosa? Please, discriminate how many were from the analyses lab and purposely collected
**Authors’ response:**

Done.

Lines 127-128 – which criteria (statistical, etc.) have determined these samples sizes?
**Authors’ response:**

See comments above.

Line 130 – delete “anamnestic” and change to: … sampling animals were clinically examined and data (i.e., age, sex, breed, clinical…
Line 140 – change to: L. tarentolae by IFAT, whereas CANINE and FELINE sera to both Leishmania spp. WERE TESTED only by IFAT.
Line 179 – change to: … 1 (ITS1, ~300bp) and AMPLIFICATION RUN AS DESCRIBED ELSEWHERE [26].
Line 186 – use CI (instead of CIs)
Line 199 – to both Leishmania spp.
**Authors’ response:**

Done

Are there any results regarding agreement between serology and PCR for humans? The same question for dogs and cats
**Authors’ response:** the agreement between serological and molecular tests has been calculated and added in the test

Line 216 – insert comma after gene,
Line 261 – Are these seronegative patients? (The detection of parasite’s DNA in few patients suggests that the parasitemia is very low or…)

Line 263 – which of the protozoa?
**Authors’ response:**

The sentence has been modified.

Line 264 – replace individuals with “humans” (in order to avoid confusion with dogs and cats)
**Authors’ response:**

Done.

Line 277 – could not this agreement be due to a lack of specificity of the serological tests between *L. infantum* and *L. tarentolae*?
**Authors’ response:**

Yes, it could be possible. The sentence has been amended accordingly.

Line 280 – Leishmania sp.

Line 281 – remove comma: … (i.e., 3.2%)
Line 284 – insert comma: … of a Brazilian mummy,
Line 291 – change to: may cause transient infections IN humans
Line 302 – delete: which had
Line 304 – change to: As far as the dog and cat populations living on the islands,
Line 307 – replace positive with “seropositive”

**Authors’ response:**

Done.

This seropositivity to both *L. infantum* and *L. tarentolae* should not be compared to seropositivity to only *L. infantum*
**Authors’ response:**

We compared the seroprevalence to *L. infantum* and *L. tarentolae* of pets in both islands since the environmental and climatic conditions for the perpetuation of both Phlebotominae (*Phlebotomus* spp. and *Sergentomyia* sp.) are almost the same.

Line 316 – “canine leishmaniosis” – is this just disease or disease plus subclinical infection? Please report in the main text
Line 318 – the meaning of “firstly retrieved” is not clear
Line 323 – Leishmania spp.
Line 324 – replace charming with “fascinating”
**Authors’ response:**

Done.

Reviewer #3: Although the work and the research question are very interesting, the methods used seem insufficient to demonstrate the hypotheses that authors wish to test. The fact that in cell cultures *L. tarentolae* can transform into amastigotes, doesn’t imply that the parasite can effectively replicate in macrophages and sustain infection in mammals. The existing evidence points rather to the transient presence of the parasite in mammals. Without this evidence, it is very risky to suggest that the results obtained through serology are indicative of immunity, much less cross-immunity.
**Authors’ response:**

We agree with this comment. We mitigated our conclusions by highlighting the limitations of the study and suggesting the need for further investigations in order to better decipher the role of this parasite in the epidemiology of human leishmaniasis and its implications in public health.
